# Prediction of individual weight loss using supervised learning: findings from the CALERIE^TM^ 2 study

**DOI:** 10.1016/j.ajcnut.2024.09.003

**Published:** 2024-09-11

**Authors:** Christina Glasbrenner, Christoph Höchsmann, Carl F Pieper, Paulina Wasserfurth, James L Dorling, Corby K Martin, Leanne M Redman, Karsten Koehler

**Affiliations:** 1 TUM School of Medicine and Health, Department of Health and Sport Sciences, Technical University of Munich, Munich, Germany; 2 Department of Biostatistics and Bioinformatics, Duke University School of Medicine, Durham, NC, United States; 3 Human Nutrition, School of Medicine, Dentistry & Nursing, College of Medical, Veterinary and Life Sciences, University of Glasgow, Glasgow, United Kingdom; 4 Pennington Biomedical Research Center, Baton Rouge, LA, United States

**Keywords:** caloric restriction, classification, humans, machine learning, modeling, obesity, pretreatment predictor, regression

## Abstract

**Background:**

Predicting individual weight loss (WL) responses to lifestyle interventions is challenging but might help practitioners and clinicians select the most promising approach for each individual.

**Objective:**

The primary aim of this study was to develop machine learning (ML) models to predict individual WL responses using only variables known before starting the intervention. In addition, we used ML to identify pre-intervention variables influencing the individual WL response.

**Methods:**

We used 12-mo data from the comprehensive assessment of long-term effects of reducing intake of energy (CALERIE^TM^) phase 2 study, which aimed to analyze the long-term effects of caloric restriction on human longevity. On the basis of the data from 130 subjects in the intervention group, we developed classification models to predict binary (“Success” and “No/low success”) or multiclass (“High success,” “Medium success,” and “Low/no success”) WL outcomes. Additionally, regression models were developed to predict individual weight change (percent). Models were evaluated on the basis of accuracy, sensitivity, specificity (classification models), and root mean squared error (RMSE; regression models).

**Results:**

Best classification models used 20–40 predictors and achieved 89%–97% accuracy, 91%–100% sensitivity, and 56%–86% specificity for binary classification. For multiclass classification, accuracy (69%) and sensitivity (50%) tended to be lower. The best regression performance was obtained with 36 variables with an RMSE of 2.84%. Among the 21 variables predicting individual weight change most consistently, we identified 2 novel predictors, namely orgasm satisfaction and sexual behavior/experience. Other common predictors have previously been associated with WL (16) or are already used in traditional prediction models (3).

**Conclusions:**

The prediction models could be implemented by practitioners and clinicians to support the decision of whether lifestyle interventions are sufficient or more aggressive interventions are needed for a given individual, thereby supporting better, faster, data-driven, and unbiased decisions.

The CALERIE^TM^ phase 2 study was registered at clinicaltrials.gov as NCT00427193.

## Introduction

The prevalence of overweight and obesity has increased drastically over the last decades, with >50% of the global population estimated to be overweight or obese by 2035 [1]. Overweight and obesity increase the risk of cardiovascular diseases, diabetes, certain types of cancers, and many other pathologies, presenting a major burden for healthcare systems [2]. Different approaches to achieving weight loss (WL) exist, including lifestyle interventions, pharmacologic treatments, and surgical procedures. However, lifestyle interventions remain the first-line approach and nonmedical gold standard [3,4]. With these, average weight reductions of 7% to 10% can be achieved within the first 6 mo of treatment [5,6], after which weight typically plateaus and eventually increases again.

However, the variability of individual WL is high. For example, Dent et al. [7] showed an average WL deviation of >5.3 kg between the upper and lower 95% confidence limits at 6 mo. Therefore, it would be beneficial to forecast an individual’s WL response before starting a specific intervention to help practitioners select the most promising approach for each individual and tailor treatment strategies around individual needs. Different WL prediction models exist, such as the National Institutes of Health Body Weight Planner [8,9] or the Pennington Biomedical Research Center Weight Loss Predictor [9,10]. However, these models utilize only a small set of general variables such as age, sex, anthropometrics, and physical activity level. Although these models show good results at the group level, individual WL predictions can vary by as much as 9% and deviate substantially from observed values after as little as 2 mo of intervention [9,11,12]. Hence, it is crucial to understand individual predictors to advance personalized WL predictions and treatments. Although some factors, such as age and habitual dietary fiber intake, have already been shown to influence the individual WL response [13,14], they explain very little of the variance in individual WL [15].

Machine learning (ML) deals with algorithms that improve performance by uncovering complex patterns in large datasets [[16], [17], [18]]. Supervised learning (SL), a subdomain of ML, aims to predict a target variable based on patterns learned from a labeled training set in which the corresponding target variable is already known, allowing for direct comparison of predicted and actual outcomes. Although ML has already shown potential in different medical areas [[19], [20], [21]], to our knowledge, it has not been applied to WL prediction.

The study’s primary objective was to develop and evaluate SL models to predict individual WL responses to a defined caloric restriction (CR) intervention using only pretreatment variables. These models will help to decide before the intervention whether CR is sufficient and provide foundational results to determine if predictors can be modified before the treatment to maximize response. We used data from the CR intervention group of the comprehensive assessment of long-term effects of reducing intake of energy (CALERIE^TM^) phase 2 study, a 2-y randomized controlled trial that analyzed long-term effects of CR on aging and longevity and included a broad range of variables that might predict individual responses to CR. Primary and secondary outcomes of CALERIE^TM^ were related to energy expenditure (resting metabolic rate and body temperature), biomarkers of metabolism and inflammation, and selected physiological and psychological measures [22,23]. Our working hypothesis was that ML could be applied to predict an individual’s WL response successfully and uncover previously unidentified pretreatment predictors.

## Methods

### General approach

**FIGURE 1:**
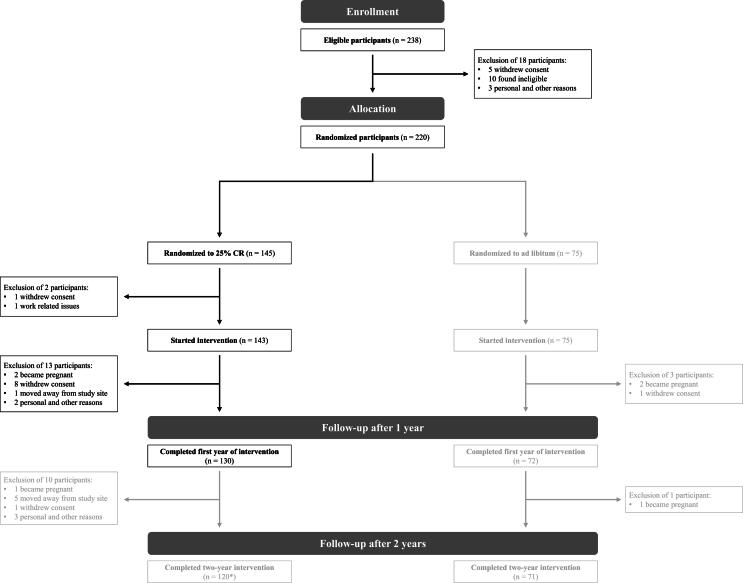
CALERIE^TM^ phase 2 Consolidated Standards of Reporting Trials (CONSORT) diagram. ∗Three additional participants were withdrawn for safety, which was not shown in the publicly available dataset. CALERIE^TM^, comprehensive assessment of long-term effects of reducing intake of energy.

The primary objective of the present study was to predict an individual’s WL response to a CR intervention using SL. For our modeling, we used data from the CR intervention group of the CALERIE^TM^ phase 2 study, which assessed >2000 different baseline variables that could potentially predict individual intervention responses [24,25]. Recruitment of the CALERIE^TM^ phase 2 study participants was conducted at the 3 clinical sites (Pennington Biomedical Research Center, Washington University Medical Center, and Tufts University) and lasted from April, 2007, to February, 2010. Participants between 21 and 50 y of age (men) or between 21 and 47 y (women) with 22.0 ≤ BMI < 28.0 kg/m^2^ were eligible for the study. Exclusion criteria included significant medical conditions, abnormal laboratory markers, psychiatric or behavioral problems, and the regular use of medications except oral contraceptives [23]. As detailed in Figure 1, 238 participants completed the baseline evaluations and were eligible for the study. Of these, 220 were assigned to the CR intervention or control group (randomly assigned, stratified by BMI, study site, and sex), and 218 started the 2-y intervention (143 in CR, 75 in control) [[26], [27], [28]]. Outcome evaluations were scheduled at baseline and after 1, 3, 6, 9, 12, 18, and 24 mo [23]. Additional details regarding the intervention design, the participant recruitment, the schedule of outcome evaluations, and the inclusion and exclusion criteria have been detailed by Rickman et al. [24], Rochon et al. [23], and Stewart et al. [26]. We used this dataset (available at calerie.duke.edu) specifically because it includes a wide array of variables that might predict individual WL intervention responses, including physiological and immune functions, physical performance, psychological outcomes, dietary information, disease risk factors, blood chemistry, and hematology [25]. Furthermore, the high quality of the dataset was ensured by a comprehensive multistep screening process to screen out ineligible volunteers [26], individual and group counseling sessions to support the study participants during the CR intervention, and closely track adherence [24], extensive database documentation [29], outcome evaluations at several predefined time points, and a study steering committee [23]. All clinical site institutions received approval from their respective institutional review boards. Participants provided written informed consent before inclusion in the trial and obtained financial compensation for their participation. The present analysis qualified for a waiver for ethical review at the Technical University of Munich, as only publicly available data were used.

Following the ML approach of Alzubi et al. [30] and Lo Vercio et al. [31], our SL workflow consisted of the following steps: First, we defined the problem and prepared the dataset for modeling. Next, we selected the features (i.e., variables used for the prediction) and chose appropriate models. Finally, we trained the models and evaluated the models’ performance. To account for the small sample size, we utilized only basic ML models, as more advanced ML models, such as deep learning algorithms, usually require a large dataset.

### Problem definition

The first step of the SL workflow consists of defining the goal of the ML model and the target variables. Our primary aim was to build SL models to predict an individual’s WL response to CR using only variables known before the start of the intervention (baseline). We used binary and multiclass classification models to predict a categorical intervention outcome of WL success and regression models to forecast relative weight change for each study participant in the CR intervention group. In addition, we wanted to identify the most essential features of each prediction model.

**FIGURE 2:**
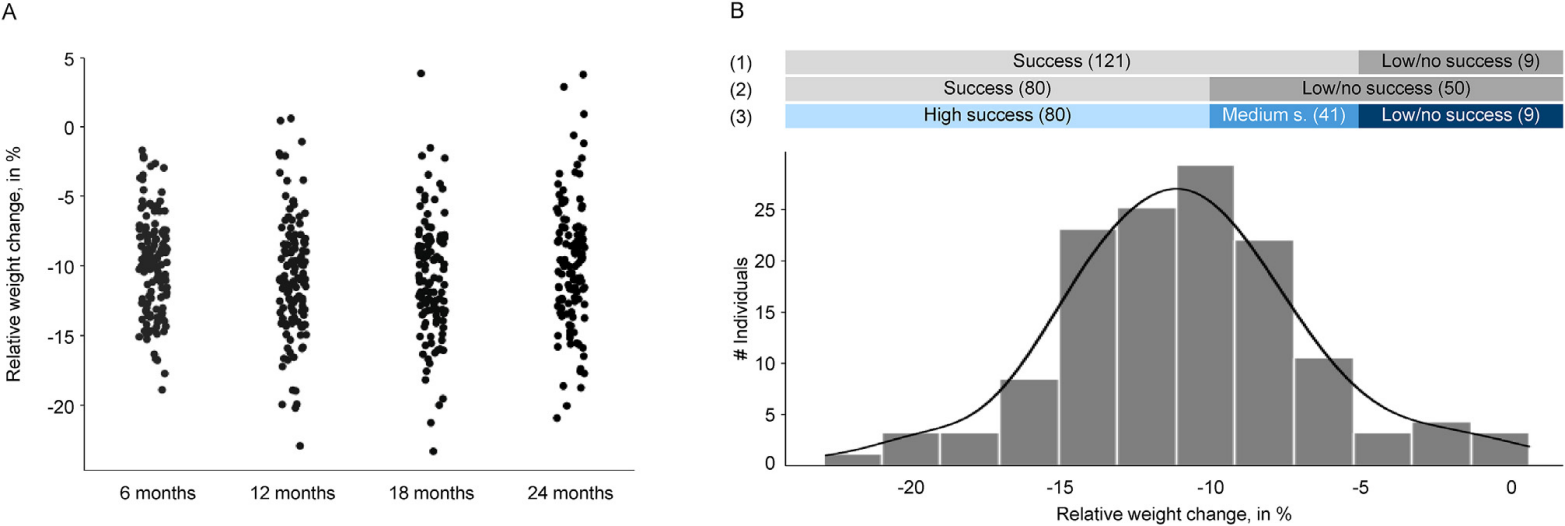
Relative weight change (A) at 6, 12, 18, and 24 mo compared with the baseline and (B) distribution at 12 mo, including the definition of classes and the number of subjects per class for binary (1–2) and multiclass (3) classification with (1) a WL threshold of 5%, (2) a WL threshold of 10%, and (3) WL thresholds of 5% and 10%. WL, weight loss.

In the CALERIE^TM^ 2 study, standardized measurements of body mass and body composition were collected using dual-energy X-ray absorptiometry (DXA), an established technique to measure body composition [32], at baseline and at 6, 12, 18, and 24 mo [23] (Figure 2A). Although the present analysis was focused on changes in body mass only and other weight variables are available in the dataset (e.g., clinical weight), we used the weight variable from DXA so that our approach could be easily modified in the future to build prediction models for more nuanced changes in body composition. Although the original study lasted 24 mo, we focused our modeling for the present analysis on weight change after 12 mo of CR, which reflected the best compromise between long-term weight change, high interindividual variability, adequate sample size, and the overall WL trajectory. As shown in Figure 2A, average WL was 7.2 ± 2.6 kg (9.94% ± 3.50%) after 6 mo and 8.0 ± 3.2 kg (10.96% ± 4.17%) after 12 mo. Weight essentially plateaued or slightly increased again at mo 18 (7.9 ± 3.3 kg; 10.91% ± 4.17%) and 24 (7.3 ± 3.5 kg; 9.98% ± 4.48%). As such, 12-mo WL represented the highest average WL of 10.96% with greater interindividual variation (4.17%) when compared with 6 mo (3.50%). Data were available for 130 subjects at 12 mo compared with 120 study participants at 18 and 24 mo, providing 10 additional data points for training a reliable model and improving the model’s generalizability [33].

**FIGURE 3:**
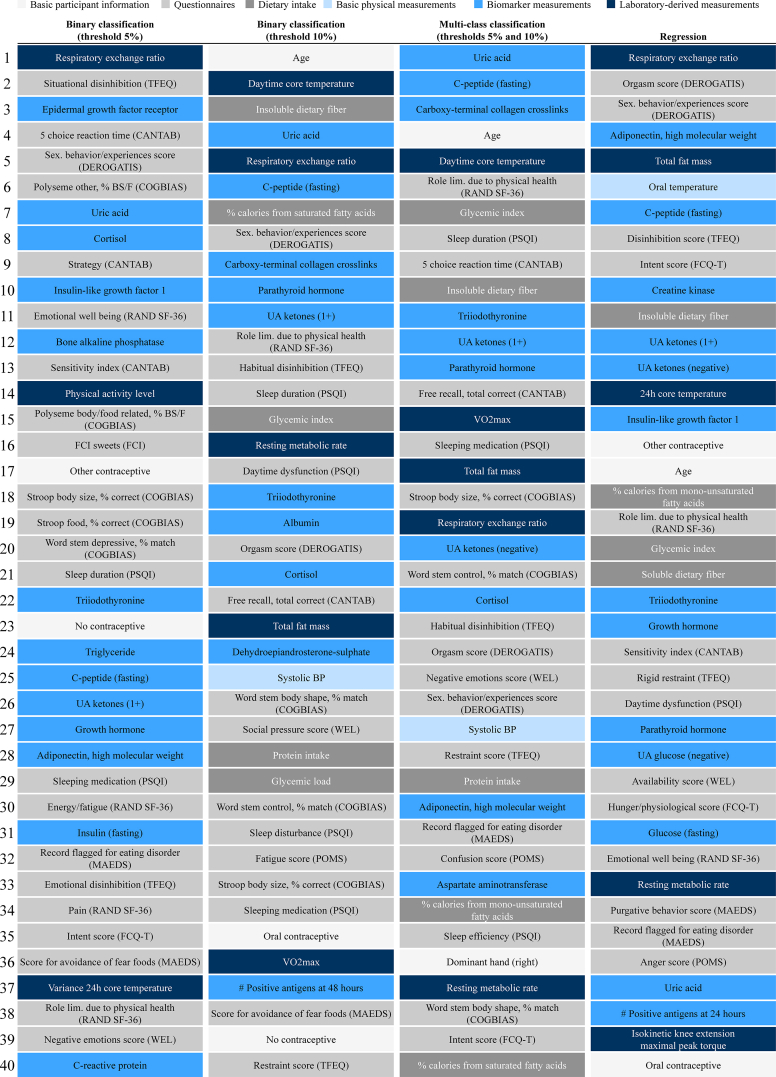
Top 40 features of each modeling task (in rank order) based on feature selection with L1 regularization, including the feature categorization in basic participant information, questionnaires, dietary intake, basic physical measurements, biomarker measurements, and laboratory-derived measurements. CANTAB, Cambridge Neuropsychological Test Automated Battery; COGBIAS, cognitive bias assessment; DEROGATIS, Derogatis interview for sexual function; FCI, food craving inventory; FCQ-T, food cravings questionnaire trait; MAEDS, multiaxial assessment of eating disorder symptoms; PSQI, Pittsburgh Sleep Quality Index; POMS, profile of mood states; RAND SF-36, RAND short form; TFEQ, three-factor eating questionnaire; WEL, weight efficacy lifestyle questionnaire.

As we wanted to illustrate classification models as an important SL technique and no categories for a discrete weight response were available in the dataset per se, we defined the target variable for classification on the basis of WL thresholds from the literature (Figure 2B). For the binary classification, we introduced the classes “Success” and “Low/no success” and considered 2 different options for the categorization: binary classification with a WL threshold of 5% at 12 mo (FIGURE 1, FIGURE 2), and binary classification with a WL threshold of 10% at 12 mo (Figure 2B, (2)). We used 5% to define “Success,” which, for people with overweight or obesity, is an established lower bound of WL to still have meaningful health benefits (e.g., reduction of cholesterol concentrations and improvement of metabolic function), as well as 10%, which is associated with additional health advantages (e.g., improvement of comorbidities, glucose metabolism, and cardiovascular disease risk factors) [[34], [35], [36]]. The 10% WL threshold is also close to the average WL of 10.96% of the study cohort after 12 mo. For multiclass classification, we introduced 3 categories (“High success,” “Medium success,” and “Low/no success”) as more classes typically increase the difficulty of the modeling task [37]. We utilized the 5% and 10% WL thresholds previously used for binary classification to define the 3 classes (FIGURE 2, FIGURE 3). For the regression, we defined the target variable as individual relative weight change (in %) at 12 mo compared with the baseline for each participant in the CR group.

### Dataset preparation

Our dataset consisted of all publicly available data from the CALERIE^TM^ phase 2 study (available through the study website calerie.duke.edu, downloaded 24 October 2022). Of the 218 study participants who started the intervention, we excluded individuals from the control group with an ad libitum diet (*n* = 75), because the purpose of our research was to predict the individual WL response to CR, and the control group underwent no CR [24]. Furthermore, we removed 13 subjects with missing data at 12 mo, resulting in a study sample of 130 participants who completed the first year of the intervention (Figure 1). The sample included in the present analysis was, on average, 38.1 ± 7.3 y old, 70% female, 168.0 ± 8.6 cm tall, weighed 72.5 ± 9.6 kg, and had a body fat percentage of 33.1% ± 6.0% (Table 1).

**TABLE 1:** Baseline characteristics of study sample.

Characteristic	Intervention group (*n* = 130)^1^
Age (y)	38.1 (7.3)
Sex, *n* (%)	
Female	91 (70)
Male	39 (30)
Height (cm)	168.9 (8.6)
Weight (kg)	72.5 (9.6)
Body fat (%)	33.1 (6.0)

1 Values are mean (SD) for continuous and *n* (%) for categorical variables.

We added the target variables defined in the Problem definition to the dataset, filtered the dataset for variables with baseline values, and removed variables with ≥25% missing values [38]. Because SL algorithms require a numeric data format as input, we transformed categorical variables into numerical ones by introducing binary dummy variables consisting of the values 0 and 1 (e.g., 1 for “Male” and 0 for “Female”). Another requirement for SL algorithms is a dataset without missing values. Therefore, we imputed the randomly missing values by assigning the mean of the respective variable, which is a standard imputation method [38]. Furthermore, we standardized the variable values to increase the comparability between the predictors. We performed both steps during the model training to avoid data leakage, meaning that the information of the test set is not used for the imputation and standardization of the training set.

### Feature selection

The initial dataset included >2000 different baseline variables. To avoid overfitting, we first screened the variables manually and removed variables that could be easily neglected, such as individual questionnaire responses when a summary score was calculated (e.g., cravings for different foods were summarized into cravings for carbohydrates, fast foods, fats, and sweets), reducing the number of baseline variables to 197. To account for the complexity and effort of measurements, these variables were categorized into 6 groups: *1*) *basic participant information* such as age and sex, *2*) *data assessed by standardized questionnaires* (e.g., Cambridge Neuropsychological Test Automated Battery), *3*) *dietary intake* (e.g., total sugars) from a food diary, *4*) *basic physical measurements* such as height and heart rate, *5*) *biomarker measurements* such as insulin, which require the collection of biological specimen and analysis in a certified laboratory, and *6*) advanced *laboratory-derived* *measurements* (e.g., resting metabolic rate), which require specific equipment. This categorization was intended to account for the effort required, as obtaining basic participant demographics is less complex and more cost effective than laboratory-derived measurements. The variables from the categories of basic participant information, questionnaires, and dietary intake can be gathered directly from the subject without help or specific measurement devices (e.g., questionnaires can be conducted online, or the individual can document dietary intake). In contrast, variables from the other categories can only be measured by a healthcare professional or an individual using simple portable devices (e.g., a heart rate monitor). A complete overview of the 197 features and the respective categories is provided in Supplemental Table 1.

As a second step in dimensionality reduction, we applied L1 regularization to select the most relevant features for modeling from the complete feature set. L1 regularization is an extension of standard regression, which penalizes a model for increasing complexity by adding a penalty term, based on the L1 norm of the coefficient vector, to the loss function. L1 regularization shrinks the coefficients of nonimportant features to 0, which can then be removed from the model. If variables are correlated, L1 regularization chooses only 1 among them and shrinks the coefficients of the others to 0. When using scaled features, coefficients with a larger absolute value correspond to variables with a higher impact on the target variable [17,[39], [40], [41]].

### Model selection

Many different algorithms exist in the literature for classification and regression tasks, including linear and nonlinear models, classification/regression trees, and rule-based models [17]. We focused on the standard models for SL [31,42], including the k-nearest neighbors model (KNN) and logistic regression for classification. The KNN algorithm assigns the most common label of the *k* most similar observations in the training set, where *k* is a natural number representing the number of neighbors. Logistic regression is a classification model, even if the name suggests otherwise, that predicts the probability that an observation belongs to a given class or not. We used linear regression and regularized regression – as a more advanced method – for the regression. We considered L1 regularization (Lasso), which adds the absolute value of the magnitude of coefficients (L1 norm) as a penalty term to the loss function, and L2 regularization (Ridge), which, as a key difference between these methods, adds the squared magnitude of coefficients (L2 norm) as a penalty term.

### Model training

We split the dataset into training and test sets to evaluate the model’s performance on new, unseen data. Because our dataset consisted only of 130 subjects from the CR intervention group, we applied leave-one-out cross-validation, which selects 1 subject of the dataset and trains a model using all other subjects. The model is then tested on the left-out subject (test set). This step is repeated until every subject is once chosen as a test set, and the performance measures from each iteration are aggregated [17]. Finally, we fitted the model to the training data (i.e., the model learned patterns from the training set).

### Performance evaluation

The final step of the SL workflow is evaluating the model’s performance. For this, we used standard performance metrics for SL, which can be found in Table 2 [17,43,44].

**TABLE 2:** Standard performance metrics for supervised learning.

Modeling task	Performance metric	Interpretation
Binary classification	Confusion matrix	*The diagonal shows cases with correctly predicted classes, and the off-diagonal illustrates the number of errors for each class.*
	$Accuracy=\frac{TP+TN}{TP+TN+FN+FP}$	*Accuracy is the ratio between all correct predictions and the total number of observations.*

	$Sensitivity=\frac{TP}{TP+FN}$	*Sensitivity measures the rate of positive cases predicted correctly (TP rate).*

$Specificity=\frac{TN}{TN+FP}$	*Specificity measures the rate of negative cases predicted correctly (TN rate).*

Multiclass classification	$Accuracy=\frac{\sum_{i=1}^{n}TP_{i}}{n}$	*As in the binary case, accuracy in the multiclass case is the ratio between all correct predictions and the total number of observations.*
	$Sensitivity=\frac{\sum_{i=1}^{n}\frac{TP_{i}}{TP_{i}+FN_{i}}}{n}$	*Sensitivity in the multiclass case measures the average per-class effectiveness of a classifier to identify class labels.*

Regression	$RMSE=\sqrt{\sum_{i=1}^{n}\frac{{\left({Predicted}_{i}-{Actual}_{i}\right)}^{2}}{n}}$	*The RMSE describes the average distance between the predicted model values and the actual values. When comparing different regression models, the best model is the one with the lowest RMSE value.*

Abbreviations: FN, false negative; FN_i_, false negative for class i; FP, false positive; *n*, number of subjects in the dataset; RMSE, root mean squared error; TN, true negative; TP, true positive; TP_i_, true positive for class i.

We compared the performance of our SL models to baseline models, which is a standard practice in ML. As a benchmark, we used a model that always predicts the most frequent class of the training data for the classification tasks and the average of the training data for the regression [45,46].

For the binary classification, we considered specificity as the most crucial performance measure because we wanted to avoid as many false positives as possible. Classifying a nonsuccessful person as successful before the start of the intervention may not lead to satisfactory intervention results for this individual. Therefore, the best-performing model should have a high specificity with, at the same time, high accuracy and high sensitivity (i.e., all performance metrics should be as close to 100% as possible). In the multiclass case, the best-performing model should have high accuracy and high average sensitivity. For the regression, the best model is the model with the lowest root mean squared error (RMSE) value.

### Single-predictor analysis

To illustrate the model results and particularly the impact of single predictors, we focused on the best-performing regression model, which is generally easier to interpret than a classification model because of the linear relationships between the variables and the outcome [17]. We analyzed the direction and relative impact of changes in a single predictor on weight change predictions for an exemplary individual with average baseline characteristics, assessing whether the weight change increased or decreased after changing a single feature value. For this, we adjusted 1 feature value at baseline by ±50% after feature normalization to a 0–1 range while keeping all other feature values constant. We then compared the predicted model values before and after adjusting the feature. This procedure was limited to the most important continuous model features, as the percentage changes in a feature value are not meaningful for discrete or corresponding dummy variables.

### Implementation

We used Python [Python 3.10.1 (ipykernel); Python Software Foundation] and built our models utilizing the *scikit-learn* package (version 1.3.2) for the implementation.

## Results

### Feature selection with L1 regularization

A total of 197 baseline variables were included in the dataset as potential model features. We performed the feature selection for each modeling task using L1 regularization (Table 3).

**TABLE 3:** Number of selected features after feature selection with L1 regularization.

Modeling task	Basic participant information, *n* (%)	Questionnaires, *n* (%)	Dietary intake, *n* (%)	Basic physical measurements, *n* (%)	Biomarker measurements, *n* (%)	Laboratory-derived measurements, *n* (%)	Total, *n* (%)
Binary classification (threshold 5%)	2 (5%)	23 (55%)	0 (0%)	0 (0%)	14 (33%)	3 (7%)	42
Binary classification (threshold 10%)	4 (6%)	35 (51%)	5 (7%)	3 (4%)	16 (23%)	6 (9%)	69
Multiclass classification (thresholds 5% and 10%)	3 (6%)	18 (38%)	6 (13%)	4 (9%)	11 (23%)	5 (11%)	47
Regression	4 (6%)	30 (42%)	6 (8%)	3 (4%)	24 (33%)	5 (7%)	72

The number of features selected to predict an individual’s WL response after 12 mo of CR ranged from 42 to 69 for the classification and was 72 for the regression (Table 3). Most features were from questionnaires or biomarker measurements. The distribution of feature categories was very similar for all modeling tasks, with the greatest difference in the features from questionnaires, used predominantly for binary classification. Compared with the other modeling tasks, more laboratory-derived measurements were used for multiclass classification.

Figure 3 displays the 40 most relevant features for each modeling task. The following 21 features were used for (almost) all modeling tasks, indicating their substantial impact on individual weight change: age (basic participant information); eating disorder flag, intent score, orgasm satisfaction, limitations due to health, sexual behavior/experiences, sleep duration, sleep medication, and Stroop body size (questionnaires); insoluble dietary fiber and glycemic index (dietary intake); adiponectin, cortisol, C-peptide, ketones, parathyroid hormone, triiodothyronine, and uric acid (biomarker measurements); and body fat, respiratory exchange ratio, and resting metabolic rate (laboratory-derived measurements).

### Performance evaluation

**FIGURE 4:**
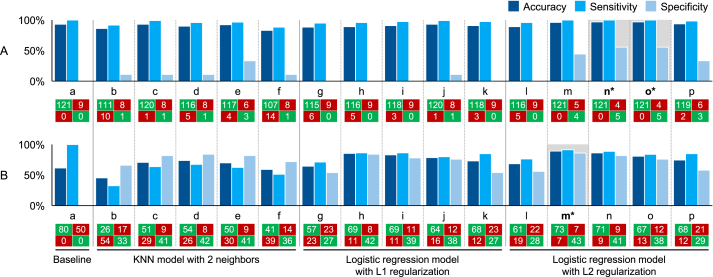
Model performance metrics (accuracy, sensitivity, specificity, and confusion matrix) for the binary classification using (A) a WL threshold of 5% and (B) a WL threshold of 10% for (a) a baseline model, (b–f) a KNN model with 2 neighbors (with (b) all features, (c) top 40 features, (d) top 30 features, (e) top 20 features, and (f) top 10 features), (g–k) a logistic regression model with L1 regularization (with (g) all features, (h) top 40 features, (i) top 30 features, (j) top 20 features, and (k) top 10 features), and (l–p) a logistic regression model with L2 regularization (with (l) all features, (m) top 40 features, (n) top 30 features, (o) top 20 features, and (p) top 10 features). ∗marks the models with the best overall performance (highest accuracy, sensitivity, and specificity). KNN, k-nearest neighbors; WL, weight loss.

The best performance of the binary classification (Figure 4) with a WL threshold of 5% was achieved with a logistic regression model with L2 regularization using only the top 20 features (97% accuracy, 100% sensitivity, and 56% specificity; features ranging from respiratory exchange ratio to word stem depressive). We received the same result with the top 30 features (features from respiratory exchange ratio to energy/fatigue), indicating that the additional 10 features did not improve performance. The best-performing model for the 10% WL threshold was also the logistic regression with L2 regularization using the top 40 features (89% accuracy, 91% sensitivity, and 86% specificity).

**FIGURE 5:**
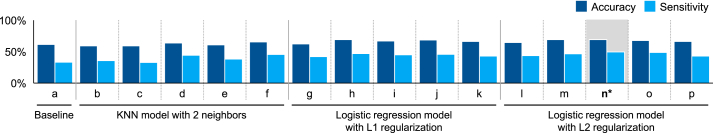
Model performance metrics (accuracy and sensitivity) for the multiclass classification for (a) a baseline model, (b–f) a KNN model with 2 neighbors (with (b) all features, (c) top 40 features, (d) top 30 features, (e) top 20 features, and (f) top 10 features), (g–k) a logistic regression model with L1 regularization (with (g) all features, (h) top 40 features, (i) top 30 features, (j) top 20 features, and (k) top 10 features), and (l–p) a logistic regression model with L2 regularization (with (l) all features, (m) top 40 features, (n) top 30 features, (o) top 20 features, and (p) top 10 features). ∗ marks the models with the best overall performance (highest accuracy and sensitivity). KNN, k-nearest neighbors.

For the multiclass classification based on the 5% and 10% WL thresholds (Figure 5), the best performance was obtained with a logistic regression model with L2 regularization using the top 30 features (69% accuracy and 50% sensitivity; features from uric acid to adiponectin).

**FIGURE 6:**
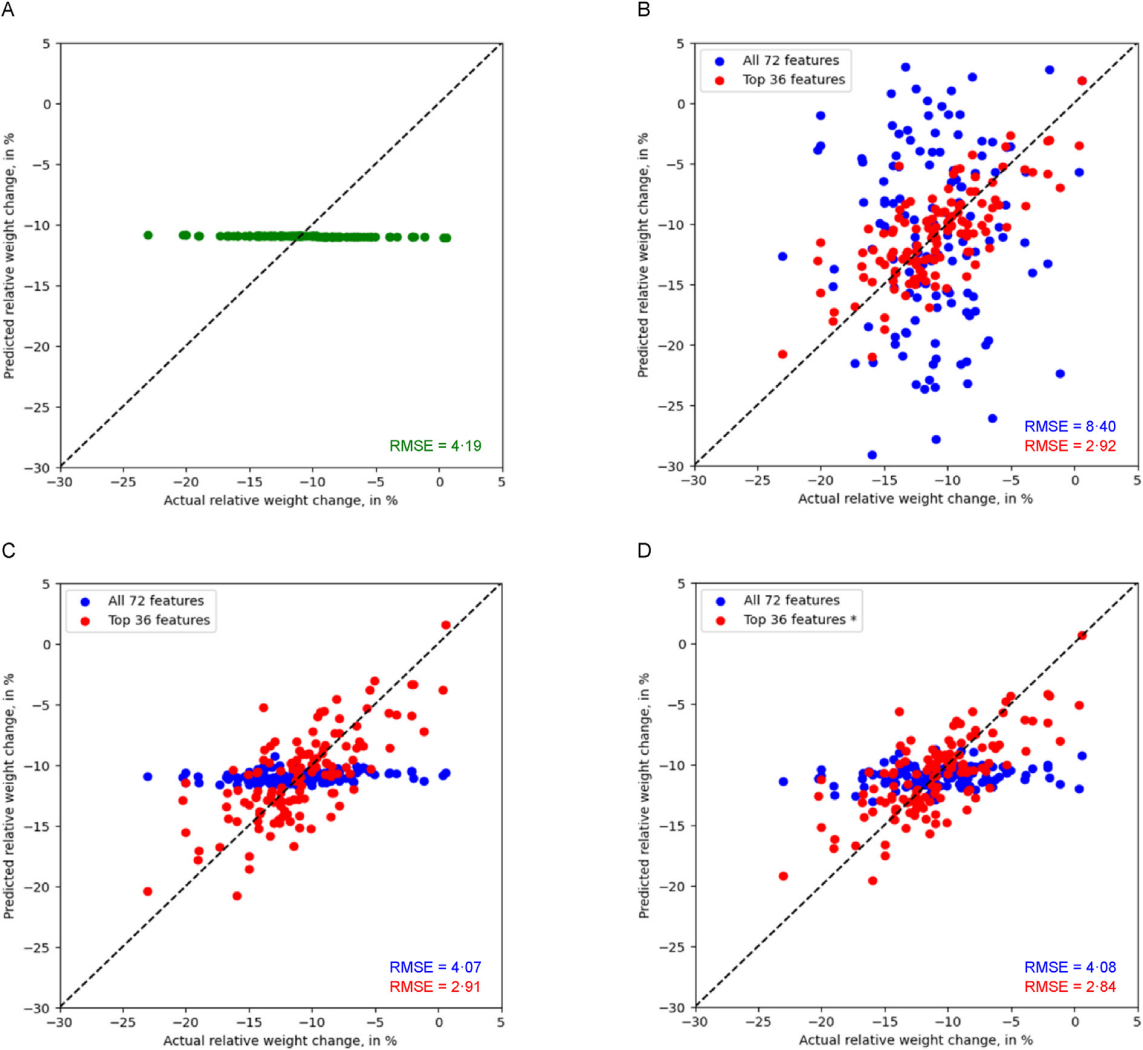
Model performance metric (root mean squared error) for the regression for (A) a baseline model, (B) a linear model without regularization (all 72 features versus top 36 features), (C) a linear model with L1 regularization (all 72 features versus top 36 features), and (D) a linear model with L2 regularization (all 72 features versus top 36 features). At the diagonal, the actual and predicted values coincide; ∗ marks the model with the best performance (lowest root mean squared error).

The performance of the regression models is illustrated in Figure 6. Each regression model variant performed best using the top 36 features. The overall best performance with an RMSE value of 2.84% was obtained with a linear model with L2 regularization (Figure 6).

### Single-predictor analysis

**FIGURE 7:**
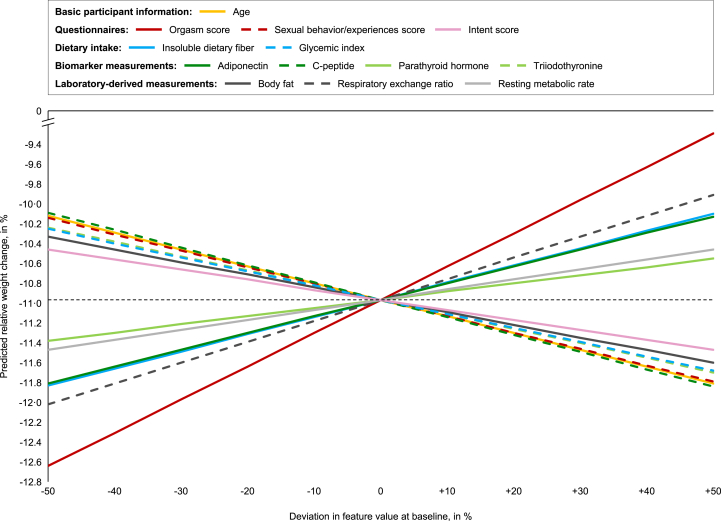
Predicted relative weight change after 12 mo of CR for deviations in single feature values at baseline for a single individual based on a linear model with L2 regularization using the top 36 features. Only the top 21 identified features were analyzed, except 5 variables (cortisol, sleep duration, sleep medication, Stroop body size, and uric acid), which were not used in the regression model, and 3 variables (eating disorder flag, ketone levels, and limitations due to health), which are discrete variables; the deviation in the feature value at baseline was only considered within the range of each single feature; the horizontal dashed line shows the predicted relative weight change for the selected subject (10.97% WL) without any deviation in the feature value at baseline. CR, caloric restriction; WL, weight loss.

The impact on relative weight change of single predictors was analyzed for an exemplary individual using the top 21 features with the exception of 5 variables (cortisol, sleep duration, sleep medication, Stroop body size, and uric acid), which were not used in the regression model, and 3 variables (eating disorder flag, ketone concentrations, and limitations due to health), which are discrete variables, using the linear model with L2 regularization (Figure 7). Higher baseline values for the variables adiponectin, insoluble dietary fiber, orgasm satisfaction, parathyroid hormone, respiratory exchange ratio, and resting metabolic rate resulted in lower predicted relative weight change at 12 mo. Conversely, greater baseline values for the features age, body fat, C-peptide, glycemic index, intent score, sexual behavior/experiences, and triiodothyronine increase weight change predictions at 12 mo. We observed the strongest impact on relative weight change for orgasm satisfaction (a 50% lower baseline value resulted in a 1.67 pp. increase in relative weight change) and respiratory exchange ratio (a 50% lower baseline value resulted in a 1.05 pp. increase in relative weight change).

## Discussion

We developed ML algorithms to predict individual WL responses after 12 mo of CR using only pretreatment variables. The best-performing algorithms were logistic/linear regression models with regularization using 20–40 features. Key predictors included basic participant information (age); questionnaire data (eating disorder flag, intent score, orgasm satisfaction, limitations due to health, sexual behavior/experiences, sleep duration, sleep medication, and Stroop body size); dietary information (insoluble dietary fiber and glycemic index); biomarkers (adiponectin, cortisol, C-peptide, ketones, parathyroid hormone, triiodothyronine, and uric acid); and laboratory-derived measurements (body fat, respiratory exchange ratio, and resting metabolic rate).

Among these, age, dietary fiber, and resting metabolic rate have already been implemented in existing WL prediction models [10,[12], [13], [14],^47^,^48^], validating our findings. Although age has previously been shown to be associated with WL success [13,14], it remains questionable whether older or younger adults lose more weight, as opposing results have been reported in the literature [13]. In our example, greater age at baseline (within the study range of 21–50 y [24]) resulted in a greater WL prediction. Furthermore, our findings suggest that a greater fiber intake attenuated WL predictions, contrary to previous reports [49,50]. In addition, our analysis revealed that a higher resting metabolic rate predicted less WL, contradicting previous studies [14,51].

A connection between WL and 16 other key features has been reported in the literature. Among laboratory-derived measurements, body fat [13,14,52,53] and respiratory exchange ratio [52] were key predictors, which is in agreement with a previous study reporting a higher respiratory exchange ratio predictive of weight gain [52,54,55]. Furthermore, we identified adiponectin [[56], [57], [58], [59]], cortisol [60], C-peptide [56], ketones [61], parathyroid hormone [62], triiodothyronine [[56], [57], [62]], and uric acid [63] as predictive biomarkers. Our results indicate that higher baseline concentrations of adiponectin and parathyroid hormone predicted less WL, in accordance with previous studies [[56], [57], [58], [59],^62^], whereas greater concentrations of C-peptide and triiodothyronine predicted more WL, contrary to a previous study [56]. For dietary intake, our models show that a higher glycemic index predicts more WL, but the impact of glycemic index on WL has been inconclusive in the literature [53,64]. From questionnaires, eating behaviors [65,66], sleep [67,68], and physical health [22,69] impact individual WL predictions.

We further identified 2 novel predictors, namely, orgasm satisfaction and sexual behavior/experiences. Although several studies have shown that WL is associated with improved sexual quality of life [70,71], the reverse direction has, to our knowledge, not yet been reported. Our results suggest that both features impact individual weight change but with opposing directionality, as higher orgasm satisfaction attenuated WL predictions, whereas greater sexual behavior/experiences (i.e., a higher frequency of sexual activities) resulted in greater WL predictions.

In summary, our findings indicate that even in a relatively healthy study sample, individuals who tended to be metabolically more unhealthy, as indicated, for example, by indicators of adiposity (body fat, adiponectin) or inflammatory markers (C-peptide) or demonstrated unhealthier dietary patterns (e.g., lower dietary fiber intake) were predicted to lose more weight during the CR intervention, although this interpretation is not consistent for all variables (e.g., triiodothyronine). Although most of our key predictors are modifiable in theory, it makes little sense to modify these parameters before treatment, which would impair metabolic health at baseline (e.g., higher body fat percentage).

### Limitations

Despite our good model performance, we acknowledge limitations of our approach. First, we only used 1 dataset. However, because CALERIE^TM^ 2 was a well-controlled clinical study with a high-quality dataset containing many variables [23], we were able to train the model on the basis of a comprehensive dataset. After model training, the algorithm can be adjusted to better meet specific real-life requirements. Second, the study sample was rather small, so we utilized basic SL algorithms applying leave-one-out cross-validation rather than more complex models, which require substantially more data [31]. Even though the inclusion of the control group would have increased our sample size, we excluded it from our analysis because we were interested in predicting the individual WL response to the intervention (CR) rather than inclusion in the study itself. Instead of CR, the control group had an ad libitum diet, which resulted in virtually no WL after 12 mo (0.23%). Nevertheless, the control group can be used to confirm whether our study sample was representative. If we assume that the control group also underwent CR, our best-performing regression model predicted an average WL after 12 mo of 10.80%, which is almost identical to the WL observed in the intervention group (10.96%). Third, we only considered the WL response at 12 mo, as it has been reported previously that CALERIE^TM^ 2 participants reached a WL plateau after roughly 60 wk [72]. As our research focused on weight reduction, we applied our ML approach only on WL after 12 mo, which was highly correlated with weight changes at other time points (data not shown). Fourth, the participants were healthy individuals meeting specific inclusion criteria (e.g., for age and BMI) [24], affecting the model’s generalizability but providing a good starting point. Fifth, the participants received intense support during CR [23], which was not accounted for in our models as we only utilized pretreatment variables. Therefore, interventions with differing levels of support should be analyzed separately.

### Future directions

Our findings demonstrate the potential of ML in forecasting individual responses to a CR intervention and identifying responders/nonresponders based on pretreatment variables. This is an example of how ML can contribute substantially to precision medicine [73], as our findings support better, faster, data-driven, and unbiased decisions. Specifically, our results propose parameters practitioners and clinicians can use to evaluate if CR is suitable for a person and to analyze if a CR intervention should be supplemented by additional personalized support to improve individual factors during the intervention (e.g., sleep duration and eating behavior) to maximize treatment response. Furthermore, this initial evaluation could guide the allocation of more intensive and expensive WL approaches (e.g., pharmacotherapy or surgeries) to those unlikely to succeed with other methods. Personalized treatment has the potential to be more effective, leading to greater WL responses and optimizing the use of resources.

Future studies should apply ML models to larger, more comprehensive datasets involving CR and other WL approaches, particularly in individuals with obesity, to assess WL prediction and identify the most effective treatment. The predictive power of our models should be tested in prospective studies and real-life applications. One important question remains whether a personalized approach, where individuals predicted to be nonresponders receive additional or more aggressive treatments (e.g., use of glucagon-like peptide receptor agonists [74]), outperforms a “one-size-fits-all” approach.

## Author contributions

The authors’ contributions were as follows – CG, KK: designed research; CG: conducted research; CH, CFP, PW, KK: provided essential materials; CG: analyzed data and built supervised learning models; CG, KK: wrote the paper; KK: had primary responsibility for the final content; KK, CH, CFP, PW, JLD, CKM, LMR: provided critical revision of the manuscript for important intellectual content; and all authors read and approved the final manuscript.

## Funding

The CALERIE^TM^ phase 2 study was supported by the National Institute on Aging and the National Institute of Diabetes and Digestive and Kidney Diseases (grants U01 AG022132, U01 AG020478, U01 AG020487, U01 AG020480, and R33 AG070455), as well as the Nutrition Obesity Research Center (grant P30 DK072476), sponsored by the National Institute of Diabetes and Digestive and Kidney Diseases; and the National Institute of General Medical Sciences of the National Institutes of Health, which funds the Louisiana Clinical and Translational Science Center (grant U54 GM104940).

## Data availability

Data described in the manuscript and analytic code will be made available upon request from the corresponding author (KK).

## Conflict of interest

The authors report no conflicts of interest.
