# Supplementary material for: Prediction of individual weight loss using supervised learning: findings from the CALERIETM 2 study
Source: Am J Clin Nutr. 2024 Sep 11;120(5):1233–44. doi: 10.1016/j.ajcnut.2024.09.003 (PMC11600119; doi:10.1016/j.ajcnut.2024.09.003)
Supplement: Multimedia component 1 [file mmc1.pdf]

Supplementary materials

Supplementary Table 1: Overview of baseline variables after manual screening.

Category	Variable name
**Basic participant information**	Age
	Dominant hand (left)
	Dominant hand (right)
	No contraceptive
	Oral contraceptive
	Other contraceptive
	Sex
**Questionnaires**	*Beck Depression Inventory (BDI)*
	BDI score
	Body Shape Questionnaire (BSQ)
	BSQ score
	Cambridge Neuropsychological Test Automated Battery (CANTAB)
	% correct simultaneous (memory, delayed matching to sample)
	5 choice reaction time (attention and psychomotor speed)
	Between errors (executive function, spatial working memory)
	Completed stage trials (executive function, intra-extra dimensional set shift)
	Free recall, total correct (memory, verbal recognition memory)
	Mean latency (rapid visual information processing)
	Sensitivity index (rapid visual information processing)
	Simple reaction time (attention and psychomotor speed)
	Strategy (executive function, spatial working memory)
	Total errors (executive function, intra-extra dimensional set shift)
	Cognitive Bias Assessment (COGBIAS)
	Polyseme body/food related, % BS/F
	Polyseme other, % BS/F
	Stroop body size, % correct
	Stroop control, % correct
	Stroop depressive, % correct
	Stroop food + body, % correct
	Stroop food, % correct
	Word stem body shape, % match
	Word stem control, % match
	Word stem depressive, % match
	Word stem food + body, % match

Word stem food, % match

Derogatis Interview for Sexual Function (DEROGATIS)

Drive and relationship score

Orgasm score

Sexual arousal score

Sexual behavior/experiences score

Sexual cognition/fantasy score

Total Derogatis score

Food Craving Inventory (FCI)

FCI carbohydrates

FCI fast foods

FCI fats

FCI sweets

Food Cravings Questionnaire State (FCQ-S)

Anticipation negative score

Anticipation positive score

Desire score

Hunger/physiological score

Lack of control score

Food Cravings Questionnaire Trait (FCQ-T)

Control score

Cues/environment score

Emotions/negative affect score

Guilt score

Hunger/physiological score

Intent score

Negative anticipation score

Positive anticipation score

Thoughts score

Multiaxial Assessment of Eating Disorder Symptoms (MAEDS)

Binge eating score

Depression score

Fear of fatness score

Purgative behavior score

Record flagged for eating disorder

Score for avoidance of fear foods

Score for restrictive eating

Perceived Stress Scale (PSS)

Perceived stress score

Pittsburgh Sleep Quality Index (PSQI)

Daytime dysfunction

Global PSQI score

Sleep disturbance

Sleep duration

Sleep efficiency

Sleep latency

Sleeping medication

Subjective sleep quality

Profile of Mood States (POMS)

Anger score

Confusion score

Depression score

Fatigue score

Tension score

Total POMS mood disturbance score

Vigor score

RAND Short Form (RAND SF-36)

Emotional wellbeing

Energy/fatigue

General health

Pain

Physical functioning

Role limitations due to emotional problems

Role limitations due to physical health

Social functioning

Three-Factor Eating Questionnaire (TFEQ)

Disinhibition score

Emotional disinhibition

External hunger

Flexible restraint

Habitual disinhibition

Internal hunger

Perceived hunger score

Restraint score

Rigid restraint

Situational disinhibition

Weight Efficacy Lifestyle Questionnaire (WEL)

Availability score

Global WEL score

Negative emotions score

	Physical discomfort score
	Positive activities score
	Social pressure score
**Dietary intake**	% calories from monounsaturated fatty acids
	% calories from polyunsaturated fatty acids
	% calories from saturated fatty acids
	Alcohol intake
	Animal protein
	Carbohydrate intake
	Energy intake
	Fat intake
	Glycemic index
	Glycemic load
	Insoluble dietary fiber
	Mean % calories from alcohol
	Mean % calories from carbohydrate
	Mean % calories from fat
	Mean % calories from protein
	Protein intake
	Soluble dietary fiber
	Total dietary fiber
	Total grams
	Total sugars
	Vegetable protein
	Water
**Basic physical measurements**	Heart rate (max)
	Height
	Mean diastolic BP
	Mean systolic BP
	Mean waist measurement
	Oral temperature
	Pulse
	Respirations (/minute)
**Biomarker measurements**	# Positive antigens at 24 hours
	# Positive antigens at 48 hours
	Adiponectin (high molecular weight)
	Adiponectin (total)
	Albumin
	Alanine aminotransferase
	Aspartate aminotransferase
	Bone alkaline phosphatase

	Cholesterol (total)
	Cortisol
	C-peptide (fasting)
	C-reactive protein
	Creatine kinase
	Creatinine
	Carboxy-terminal collagen crosslinks
	Dehydroepiandrosterone-sulphate
	Epidermal growth factor receptor
	Glucose (fasting)
	Growth hormone
	High-density lipoprotein cholesterol
	Hematocrit
	Hemoglobin
	Insulin-like growth factor 1
	Insulin (fasting)
	Insulin resistance homeostasis model assessment
	Insulin sensitivity
	Interleukin 6
	Low-density lipoprotein cholesterol
	Leptin
	Lymphocytes (absolute)
	pH
	Parathyroid hormone
	Serum glucose
	Thyroid stimulating hormone
	Tumor necrosis factor (alpha)
	Tartrate-resistant acid phosphatase 5b
	Triglyceride
	Triiodothyronine
	UA glucose (negative)
	UA ketones (1+)
	UA ketones (negative)
	UA ketones (trace)
	Uric acid
**Laboratory-derived measurements**	Average 24h core temperature
	Average daytime core temperature
	Average nighttime core temperature
	Composite strength score peak torque
	Isokinetic knee extension maximal peak torque
	Isokinetic knee flexion maximal peak torque
	Left and right leg lean mass

Peak force (left hand)

Peak force (right hand)

Physical activity level

Peak VO2

Respiratory exchange ratio

Resting metabolic rate

Total energy expenditure

Total fat mass

Total fat-free mass

Total mass

Variance 24h core temperature

Variance daytime core temperature

Variance nighttime core temperature

VO2max

Work fatigue index
